# In vitro osteoclastogenesis in autoimmune diseases – Strengths and pitfalls of a tool for studying pathological bone resorption and other disease characteristics

**DOI:** 10.1016/j.heliyon.2023.e21925

**Published:** 2023-11-03

**Authors:** Patrik Skubica, Marketa Husakova, Pavlina Dankova

**Affiliations:** a Faculty of Science, Charles University, Prague, Czech Republic; b First Faculty of Medicine, Charles University, Prague and Institute of Rheumatology, Prague, Czech Republic

**Keywords:** Osteoclast, In vitro osteoclastogenesis, Autoimmunity, Rheumatic disease, Enteropathy

## Abstract

Osteoclasts play a critical role in bone pathology frequently associated with autoimmune diseases. Studying the etiopathogenesis of these diseases and their clinical manifestations can involve *in vitro* osteoclastogenesis, an experimental technique that utilizes osteoclast precursors that are relatively easily accessible from peripheral blood or synovial fluid. However, the increasing number of methodical options to study osteoclastogenesis *in vitro* poses challenges in translating findings to clinical research and practice. This review compares and critically evaluates previous research work based on *in vitro* differentiation of human osteoclast precursors originating from patients, which aimed to explain autoimmune pathology in rheumatic and enteropathic diseases. The discussion focuses primarily on methodical differences between the studies, including the origin of osteoclast precursors, culture conditions, and methods for identifying osteoclasts and assessing their activity. Additionally, the review examines the clinical significance of the three most commonly used *in vitro* approaches: induced osteoclastogenesis, spontaneous osteoclastogenesis, and cell co-culture. By analyzing and integrating the gathered information, this review proposes general connections between different studies, even in cases where their results are seemingly contradictory. The derived conclusions and future directions aim to enhance our understanding of a potential and limitations of *in vitro* osteoclastogenesis and provide a foundation for discussing novel methods (such as osteoclastogenesis dynamic) and standardized approaches (such as spontaneous osteoclastogenesis) for future use in autoimmune disease research.

## Highlights

• *In vitro* osteoclastogenesis reflects etiopathogenesis of autoimmune diseases.
• Seemingly contradictory or scattered results make its practical use problematic.
• Differences may be explained by broad palette of approaches and methodical options.
• Certain features of osteoclastogenesis can distinguish patients from controls.
• Novel or overlooked approaches and methods promise use in precision medicine.

## 1 Introduction

Osteoporosis or osteopenia are relatively well-known consequences of chronic inflammation. Among patients suffering from long-term immune activation, the connection between decreased bone mineral density and inflammation has so far been most studied in rheumatic diseases, such as rheumatoid arthritis (RA), psoriatic arthritis (PsA), systemic lupus erythematosus (SLE) and ankylosing spondylitis (AS) [1], or enteropathic diseases, such as celiac disease (CD) [2] or Crohn's disease (CRD) [3]. Previous research has shown that the development of osteoporosis is closely linked to dysregulated osteoclastogenesis – the ability of mononuclear precursors to differentiate into osteoclasts (OC), macrophage-like multinuclear cells that specialize in resorption of bone matrix. Changes in OC behavior such as an increase in their activity were also often observed [4]. In 2006, the theory of connection between chronic inflammation and osteoporosis gained support with findings of Komano et al. [5], who identified a subset of peripheral blood CD16^−^ monocytes that can differentiate into osteoclasts. This shows that OC precursors can be affected not only in bone marrow, but also directly in the bloodstream, where long-term immune activation arises in chronic inflammatory conditions.

It is now generally accepted that inflammatory driven OC differentiation and/or activation of OC represents the main culprit between osteoporosis and rheumatic diseases [6] and one of the possible mechanisms of higher osteoporosis risk in enteropathies [2,3]. Changes in OC biology are also recognized as an important factor in several other diseases, including for example multiple myeloma [7], Paget's disease of bone [8] or periodontal disease [9]. This review will focus solely on the OC involvement in autoimmune diseases.

Alterations in several cytokines, including the osteoblasts-produced receptor activator of nuclear factor kappa B ligand (RANKL), which signals via receptor activator of nuclear factor kappa B (RANK) located on OC precursors to activate the differentiating pathway [10], have been identified as important factors leading to altered differentiation and activity of OC (previously reviewed e.g. by Lee and Lorenzo [11] or Amarasekara et al. [12]). Next to the signaling molecules participating in OC genesis and action, another extensively studied cause of osteoclast-induced osteoporosis is the osteoclastogenic capacity of the precursors as such, which can vary between healthy and diseased conditions. Despite the expectation of higher numbers of *in vitro* generated osteoclasts, it has been shown in some cases that peripheral blood monocytes (PBM) derived from diseased subjects (e.g. RA or AS) differentiate into similar or even lower numbers of OC than PBM from healthy individuals [[13], [14], [15], [16], [17]]. However, the results of studies can differ for the same disease (e.g. Im et al. [18] vs. Perpétuo et al. [15] for AS; Ikić et al. [19] vs. Marton et al. [20] for PsA or RA or Nakano et al. [21] and Hase et al. [22] vs. Luukkonen et al. [23] for RA). Similarly, some authors report significant correlations between *in vitro* OC numbers and clinical markers or disease scores [17,[24], [25], [26], [27]] while others observe no correlation [18,19,28].

Although discrepancies outlined above may come from the application of various research methods, it cannot be ruled out that they point to underlying biological mechanisms. Assessment of *in vitro* osteoclastogenesis allows examining parameters that may reflect biological processes occurring *in vivo*. These parameters include number and origin of preosteoclasts, number, morphology and viability of mature OC, their function/activity, and kinetics of the process of OC genesis. Staining for tartrate-resistant acid phosphatase (TRAP), an enzyme crucial for osteoclastic function and an established marker of OC, is considered the golden standard and also the primary requirement for further OC analyses [29]. 10.13039/100003224OC counting based on histological staining can be supported by TRAP detection in culture media by the enzyme-linked immunosorbent assay or quantification of 10.13039/100003224OC proteins or genes expression. Predominantly is the OC activity evaluated by bone resorption assay; rather rarely applied are the assessments of number of nuclei per osteoclast, area per osteoclast or number of resorption pits.

Three main approaches may be employed to study osteoclastogenesis *in vitro* – induced osteoclastogenesis, spontaneous osteoclastogenesis and cell co-culture. In the induced osteoclastogenesis, mononuclear cells are cultured in medium containing exogenously added RANKL and usually also macrophage colony-stimulating factor (M-CSF), regardless of whether fetal bovine or human serum is used in the culture. Spontaneous osteoclastogenesis, on the other hand, assumes that mononuclear cells used for cell culture already come pre-stimulated from the internal environment of the body and therefore, they do not need further artificial stimulation with RANKL. Alternatively, bodily fluids act as stimulants themselves, when added to medium – such cell culture is then performed with medium without artificially added cytokines. Lastly, in a co-culture based model, stimuli-producing cells such as T-cells, B-cells and fibroblasts or conditioned medium from simultaneous culture of these cells are added. The idea here is that other present cell types can act as secondary stimulants of OC differentiation by producing OC differentiating cytokines, especially RANKL [[30], [31], [32], [33], [34]].

Such a diverse range of applicable approaches and methods leads to the need to understand both strengths and pitfalls of each of them in order to be able to choose the proper experimental setup and correctly interpret the results. These topics become even more trending when considering that predictive models for bone resorption related diseases could be constructed based on *in vitro* osteoclastogenesis course and/or outcome [35,36]; or, it might be even possible to use it for osteoporosis screening [37]. In yet another area of research, osteoclastogenic experiments show great potential: using expression quantitative trait loci data from human osteoclasts generated *in vitro*, the functionality of genetic variants revealed in genome-wide association studies can be tested [38,39].

The main goal of this review, using evidence from previous research, is to point to overlooked aspects of *in vitro* osteoclastogenesis with respect to autoimmune diseases. Attention will be paid to enteropathic diseases CD and CRD and rheumatic diseases RA, SLE and the seronegative spondyloarthropathies, which include AS, reactive arthritis (formerly known as Reiter's syndrome), the arthritis of inflammatory bowel disease, juvenile onset spondyloarthropathy and PsA. We will show that the plethora of various methods and three main approaches used when studying osteoclastogenic capacity and OC activity can lead to some interesting findings that could be further utilized in the future research of bone pathology in these diseases.

## 2 Literature search

**Fig. 1:**
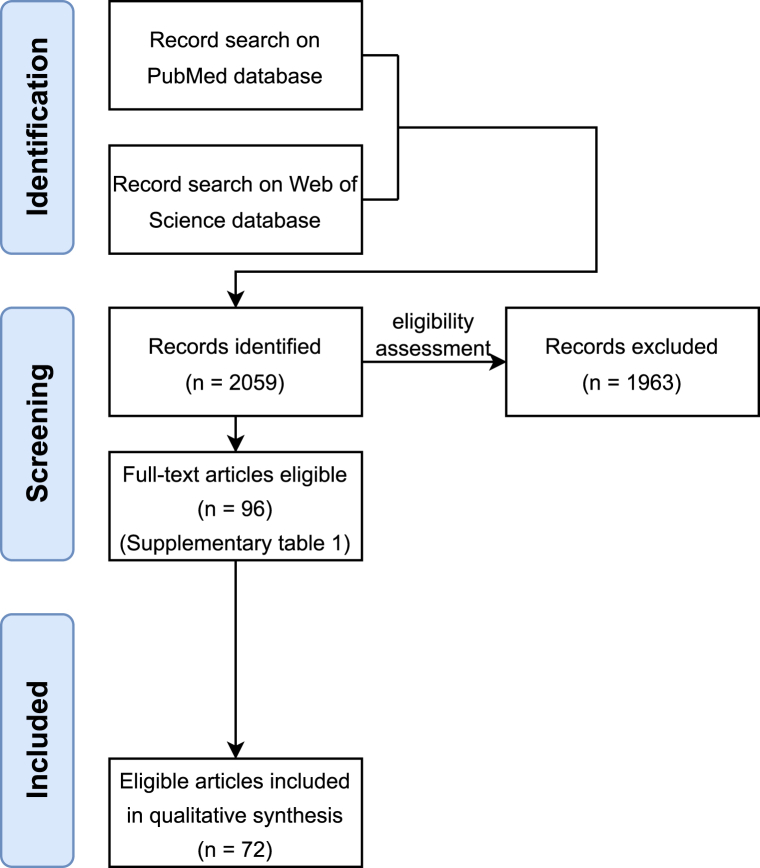
Flowchart of literature search and selection.

The electronic search for relevant studies was carried out using the PubMed database and Web of Science database. Firstly, PubMed search for articles published between time range 1999–2022 using terms (osteoclasts OR osteoclast formation OR osteoclast differentiation OR osteoclastogenesis) AND (monocytes OR mononuclear cells OR peripheral blood) AND (disease OR arthritis OR osteoporosis OR spondylitis) was performed. Then, a similar search query was used on Web of Science, which yielded comparable results with only a handful of articles not previously found on PubMed (Fig. 1).

## 3 Selection of papers

In order to select relevant studies, titles and abstracts of close to 2050 articles were manually screened. When the suitability of an article could not be decided based on title or abstract, either material and methods section or full text was read. Only research articles fulfilling all the following criteria were included.

(1) *In vitro* osteoclastogenesis was performed
(2) Human mononuclear cells from peripheral blood, bone marrow or synovial fluid were used for osteoclast differentiation
(3) Biological material from patients (cells, fluids or isolates from fluids) suffering from RA, PsA, AxSpA, AS, SLE, CD or CRD was used

Each article also had to fulfill at least one of the following criteria.

(4) Osteoclastogenesis on peripheral blood, bone marrow or synovial fluid mononuclear cells from patients with one of the diagnoses mentioned above was performed
(5) Osteoclastogenesis on peripheral blood, bone marrow or synovial fluid mononuclear cells from healthy donors (HD) was carried out and biological material (cells, fluids or isolates from fluids) from patients with one of the diagnoses mentioned above was used during culture of healthy cells

Duplicate articles and articles not in English were not included. From all original research papers that could be accessed, a total of 96 articles were selected for this study and subjected to a full analysis (see Supplementary Table 1 for a complete list of selected articles, including an overview of used methods, materials and main findings).

## 4 Methods of *in vitro* osteoclastogenesis

Results of experimental research may be influenced by relatively simple differences in experimental methods, such as the initial steps in preosteoclast stimulation or eventual evaluation of osteoclastogenesis: i) starting numbers of precursors used *in vitro* can differ considerably between studies; ii) cell types of three different origins are frequently used as OC precursors, with the most common being PBM due to their availability, followed by synovial fluid monocytes (SFM) and lastly bone marrow-derived monocytes (BMM); iii) concentrations of exogenous M-CSF and RANKL used for OC precursors priming vary broadly; and finally iv) different studies evaluate the number and activity of formed OC at different time points after the start of the osteoclastogenesis process. Moreover, it turns out that the number of mature OC does not always correlate with the total OC activity. Aim of this chapter is to report on studies that investigate these aspects of *in vitro* osteoclastogenesis and show why they should not be overlooked. Above that, we discuss some less commonly used alternatives for OC identification and study of their activity or function.

### 4.1 Number and origin of osteoclast precursors

#### 4.1.1 Number of precursors

The numbers of precursors used for osteoclastogenesis *in vitro* varies greatly across studies. For example, as low as 0.015 × 10^6^ purified monocytes per cm^2^ are sometimes seeded, compared to the amount of 0.9 × 10^6^ or 1.5 × 10^6^ monocytes used in other cases. This represents a significant difference which can be as high as a hundredfold. Similarly, numbers of mononuclear cells used range from 0.03 × 10^6^ to 3 × 10^6^ (Suppl. Table 1). However, not a lot of attention has been brought to this aspect and it is unclear if and how it affects results among studies. Findings we have so far are rather methodical, such as the need for a minimum number of precursors to differentiate into osteoclasts *in vitro* [13]. This is probably related to the recent discoveries that osteoclast precursors called “founders”, which represent only a small fraction of precursors, are able to initiate fusion into multinuclear osteoclasts after prolonged stimulation with RANKL. In contrast, the majority of precursors called “followers” cannot fuse with each other; they fuse only together with founders, even without prolonged stimulation with RANKL [40]. Alterations in this interaction are linked directly to enhanced bone resorption *in vivo* [41]. Studies on this topic involving autoimmune diseases have not yet been conducted, and the new findings may facilitate a breakthrough in the field of inflammatory osteoclastogenesis.

#### 4.1.2 Origin of precursors

Osteoclast precursors are recruited from PBM, SFM or BMM. From the biological perspective, these cell types have quite different properties and relations with respect to bone. Cancellous bone tissue is subject to more dynamic remodeling than cortical bone [42]. Like bone marrow, cancellous tissue is located in the inner parts of bone, therefore it is readily accessible to BMM. SFM, on the other hand, are found in synovial fluid, which is the part of bone articulations. While the chronic inflammation of joints in arthritis suggests biological significance of SFM, it is unclear what percentage of bone resorption can be attributed to them. Lastly, PBM are good candidates for pathological bone resorption when considering that inflammation can be “transmitted” globally around the body via PBM and blood inflammatory molecules, which would also at least partly explain the connection between chronic inflammation and systemic bone loss. Primed PBM can enter bone tissue via bloodstream, and they serve as a supplementary pool of precursors for osteoclastogenesis *in vivo* [43]. Although it is still not completely understood to what extent PBM contribute to bone resorption *in vivo*, the importance of osteoclast differentiation from PBM is recognized across wide range of chronic inflammatory conditions [44]. The osteoclastogenic potential of PBM is linked directly to bone erosions in arthritis [45] and simultaneous changes in abundance of highly pro-osteoclastogenic populations among PBM and BMM precursors have been reported in chronic inflammatory diseases [46,47].

*In vitro* studies performed on inflammatory rheumatic disease cells, however, point to different abilities of PBM and SFM in pathological osteoclastogenesis: Ikić et al. [19] showed that induced differentiation of RA PBM into osteoclasts is more than two times effective than from PBM of PsA, but it is not the case for SFM – differentiation of RA SFM yielded similar numbers of OC as PsA SFM differentiation. Interestingly, OC from PsA SFM showed some morphological differences with higher nuclei-to-cell ratio. In PsA, spontaneous differentiation of both PBM and SFM occurs; after T-cell depletion, however, SFM retain 30 % of osteoclastogenic capacity while differentiation in PBM diminishes completely [30]. This would suggest that monocytes coming from PsA synovial fluid retain relatively high osteoclastogenic capacity *in vitro* even without direct stimulation from cells and/or bodily fluids. Further findings by Mori et al. [32] show that differentiation into OC from PBM and SFM of PsA patients is blocked both by RANKL antagonist RANK-Fc and by inhibitor of tumor necrosis factor α (TNF-α), anti-TNF-α. However, while RANK-Fc has about the same efficiency as anti-TNF-α when used on PBM, anti-TNF-α is highly more efficient when used on SFM. Blood precursors thus seem to be more prone to RANKL stimulation while TNF-α stimulation appears to be critical for precursors from inflamed joints.

Even among the so far discussed OC precursors themselves, there may be specific subtypes that play a more significant role in osteoclastogenesis than others. CD11c^+^ myeloid cells in RA are epigenetically programmed towards higher *RANK* expression, and in terms of OC generation and activity they react differently to simultaneous RANKL and TNF-α stimulation when compared to CD14^+^ monocytes and moreover, they are unresponsive to TNF-mediated inhibition [48]. CD14^+^CD16^−^ PBM from RA subjects were shown to differentiate into more OC when compared to CD14^+^CD16^−^ PBM originating from HD, while there was no difference in osteoclastogenic potential of CD14^+^CD16^+^ monocytes [49]. The CD14^+^CD16^−^ monocytes were also prone to specific inhibition of tyrosine kinase Tyro3TK, which completely abolished their differentiation [49]. In AS, there was no difference in CD14^+^ and CD16^+^ monocyte counts between patients and HD, but patients’ monocytes were less likely to differentiate into OC [16]. Lastly, in RA, immature dendritic cells and myeloid-derived suppressor cells can develop into OC. The first mentioned are closely linked to anti-citrullinated protein antibody-positive RA [50], the second to highly active erosive RA [51]. Taken together, linking the specific subset of cells to changes in overall osteoclastogenic potential might prove to be particularly useful in therapy of rheumatic diseases.

### 4.2 Cytokine concentrations in *in vitro* osteoclastogenesis

**Fig. 2:**
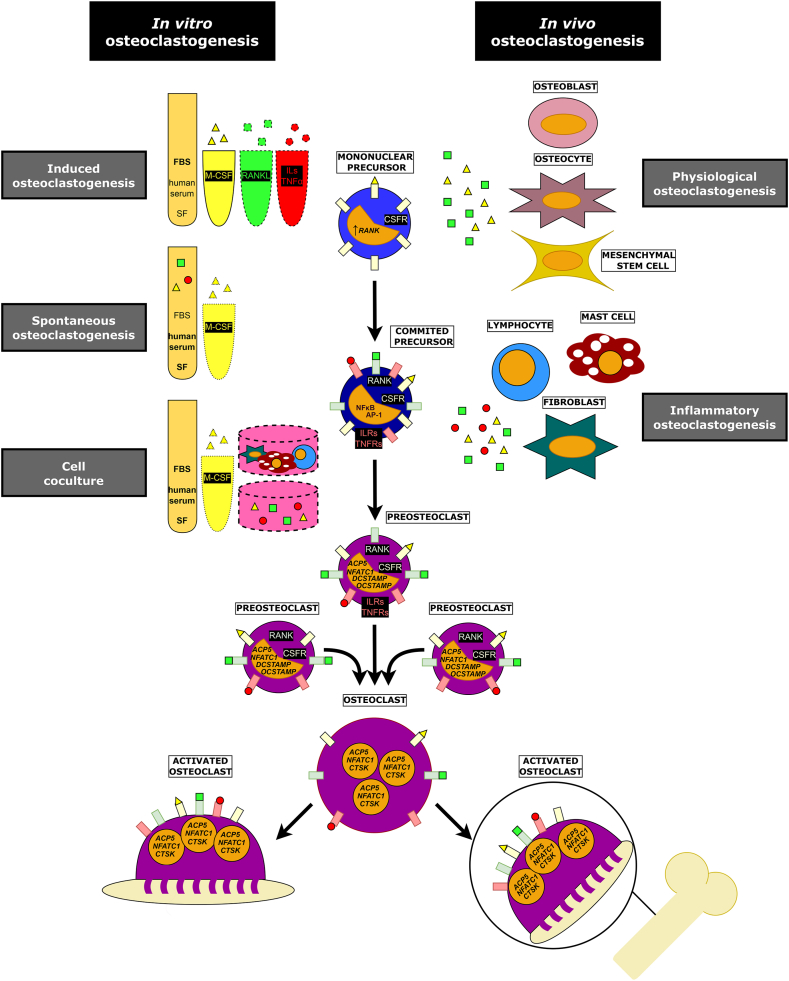
Differentiation of osteoclasts *in vitro* and *in vivo*. OC form from mononuclear precursors. After stimulation of precursor with M-CSF via CSFR, the RANK expression is initiated leading to exposure of RANK receptor on the cell membrane of committed precursor. Binding of RANKL to RANK activates the signaling cascade involving MAP kinases, NFκB and AP-1. Subsequent amplification of *NFATC1* initiates expression of early OC genes including *ACP5* and fusogenes *DCSTAMP* and *OCSTAMP*, and therefore change to TRAP positive preosteoclast. Several preosteclasts fuse to form multinuclear OC with high expression of genes coding for resorption enzymes, including *ACP5* and *CTSK* (*in vitro*, the fusion process is started by a founder cell with high expression of the fusogenes). The resulting OC are then activated into resorbing cells with characteristic membrane domains by contact with resorbable surface, which is bone tissue *in vivo* or represented by bone/dentin discs and calcium phosphate coated plastics *in vitro*. Physiologically, osteoclastogenesis is supported by osteoblasts, osteocytes or mesenchymal stem cells that produce M-CSF and RANKL. In chronic inflammatory states, this role can be adopted by other cell types, such as lymphocytes, fibroblasts, and mast cells, which in addition to M-CSF and RANKL can produce other molecules involved in osteoclastogenesis, such as ILs and TNF-α, recognized by their respective receptors on the surface of precursor/OC. *In vitro*, the role of these cells can be substituted by artificial addition of exogenous M-CSF and RANKL or other cytokines in so called induced osteoclastogenesis. Another approach, spontaneous osteoclastogenesis, assumes that osteoclastogenic stimuli are present in bodily fluids or that isolated precursors come already pre-stimulated by these stimuli; for differentiation, therefore, only serum or synovial fluid is sufficient, and M-CSF may or may not be added. In co-culture, cell types known or supposed to be involved in osteoclastogenesis, or media containing osteoclastogenic factors from separate cultures of these cells are used in OC differentiation. **Symbols:** solid line: factor always added into cell culture; dashed line: at least one of the factors added into cell culture; dotted line: factor which may/may not be added into cell culture. **Abbreviations:***ACP5*: acid phosphatase 5, tartrate resistant, gene; AP-1: activator protein 1; CSFR: macrophage colony-stimulating factor receptor; *CTSK*: cathepsin K gene; *DCSTAMP*: dendrocyte expressed seven transmembrane protein coding gene; FBS: fetal bovine serum; ILRs: interleukin receptors; ILs: interleukins; M-CSF: macrophage colony-stimulating factor; *NFATC1*: nuclear factor of activated T-cells, cytoplasmic 1, gene; NFκB: nuclear factor kappa B; *OCSTAMP*: osteoclast stimulatory transmembrane protein coding gene; OC: osteoclasts; RANK: receptor activator of nuclear factor kappa B; RANKL: receptor activator of nuclear factor kappa Β ligand; SF: synovial fluid; TNFRs: tumor necrosis factor alpha receptors; TNF-α: tumor necrosis factor alpha; TRAP: tartrate-resistant acid phosphatase.

*In vivo*, OC form from mononuclear cells under the effect of M-CSF and RANKL. Signaling of M-CSF via colony stimulating factor 1 receptor induces RANK expression in 10.13039/100003224OC precursors [52] and supports proliferation, fusion, and initiation of resorptive activity [53]. RANKL, after binding to its receptor RANK, activates a crucial signaling cascade leading to rapid activation of nuclear factor kappa B (NFκB), activator protein 1 and map kinases. Subsequent amplification of the nuclear factor of activated T-cells cytoplasmic 1 initiates the expression of OC genes and associated change to OC phenotype [54]. The importance of M-CSF and RANKL is underscored by the fact that, for long time, the *in vitro* osteoclastogenesis procedures were dependent on concurrent co-culture with osteoblasts/mesenchymal stem cells (MSC), which are now known to be (although not exclusive) source of these two cytokines [55] (Fig. 2).

When cytokines are artificially added to cell culture of OC precursors, we talk about induced osteoclastogenesis, while no addition of cytokines (excluding M-CSF) results in so called spontaneous osteoclastogenesis. Both of these approaches are described in detail in Fig. 2 and in the chapter “Main approaches for studying *in vitro* genesis of osteoclasts”. Importantly for this chapter, concentrations of M-CSF and RANKL added artificially to cell culture may differ considerably (Suppl. Table 1). M-CSF concentrations range from 2 ng/ml to 100 ng/ml, with the 25 ng/ml concentration being employed most frequently. This probably stems from the *in vitro* findings that OC differentiation rises dose-dependently up until 25 ng/ml, shows no more increase between 25 and 50 ng/ml and is inhibited with concentration greater than 100 ng/ml [53]. In case of RANKL, as low as 0.5 ng/ml and as high as 100 ng/ml is used, with the concentration range 30–50 ng/ml being the most frequent (Suppl. Table 1).

Authors who investigated this aspect of *in vitro* osteoclastogenesis reached several interesting conclusions, specifically in case of concentrations lower than 30 ng/ml RANKL, referred as “suboptimal” or “low-dose”: Using exogenous RANKL concentrations within the range of 0.5–30 ng/ml, Taranta et al. [56] showed that in the presence of suboptimal 0.5 ng/ml RANKL, serum of CD patients had higher osteoclastogenic stimulatory potential on HD PBM than healthy serum, whereas under the influence of high exogenous concentration of 30 ng/ml RANKL there was no difference in stimulatory effect between CD sera and HD sera. This effect was as high as 40-fold in sera from untreated, newly diagnosed patients, and approximately 8-fold in sera obtained from treated patients following a gluten-free diet (GFD), and was likely caused by higher *in vivo* concentrations of RANKL in both CD and CD-GFD sera. Hirayama et al. [13] observed equal osteoclastogenic potential of RA and HD PBM using RANKL concentrations 5 ng/ml, 10 ng/ml or 30 ng/ml; resorption capacity, however, was heightened in RA PBM in presence of 5 ng/ml RANKL. The importance of serum RANKL concentration in CD and RA is underlined by its direct relation to disease burden: In CD, serum RANKL is higher in patients not yet on diet [56], and in RA it is a predictor of disease progression [57] and radiographic progression [58].

Regarding the importance of concentrations of RANKL added *in vitro*, another study reported stimulatory properties of interleukin (IL)-21 in OC formation when added to cultures of HD PBM in the presence of suboptimal 10 ng/ml RANKL [59]. TNF-α and IL-33 can stimulate OC generation in a RANKL-independent manner; this effect, however, is blocked by RANKL over-stimulation [48,60]. On the same note, IL-26, IL-17A and also TNF-α lead to OC differentiation in presence of low RANKL concentrations [34,61], suggesting that inflammatory cytokines take at least partial control of osteoclastogenesis in a chronic inflammatory condition. To sum up, suboptimal RANKL concentrations may be particularly appropriate when artificial stimulation towards osteoclastogenesis is necessary, but at the same time we do not want to mask the anticipated effects of biological factors that would remain hidden at the excessive doses of exogenous RANKL.

### 4.3 Evaluation of the number of osteoclasts at different time points

The OC numbers generated *in vitro* are typically evaluated by staining for TRAP; judgment of their activity is usually based on staining of resorbed pits. Both the OC numbers and resorption activity can be assessed at different time points, providing a noteworthy methodical problem leading to some discrepancies and contradictory results between studies when not taken into consideration. On the other hand, it is an excellent research tool to determine, in addition to differences in the capacity of precursors to differentiate into OC, and differences in OC activity, any variability in the dynamics of differentiation itself. These dynamics could be an important overlooked aspect with biological/medical implications.

Cell culture lengths used for OC differentiation can be briefly divided into time spans of 1–3 weeks. For simplification, time ranges described here are as follows: 1 week = 7–10 days; 2 weeks = 11–17 days; 3 weeks = 18–24 days. By far the most common lengths seen across studies are 2 or 3 weeks; 1-week culture is less common and is sometimes performed in parallel with longer culture, which allows for comparison of osteoclastogenesis dynamics.

Rapid differentiation into OC has been repeatedly observed when monocytes are cultured together with other cell types, for example with fibroblasts/fibroblast-like synoviocytes (FLS) [62,63], nurse-like cells [64,65], MSC [66] or T-cells [67]. OC formation in the presence of body fluids can also occur in as little as one week [56,68], suggesting that conditions closer to the *in vivo* situation (which is likely represented by the presence of human serum or synovial fluid) can drive OC precursors to differentiate more rapidly. Interestingly, variable rate of differentiation of precursors into 10.13039/100003224OC may be caused by specific biology of a particular disease, because both of the last mentioned studies found noteworthy differences in dynamics of 10.13039/100003224OC generation depending on whether HD or patients’ serum was applied: Inflammatory serum of newly diagnosed 10.13039/100011639CD patients (who had not yet adhered to the gluten-free diet) supported formation of 10.13039/100003224OC from HD PBM as soon as after 7 days in culture. On the contrary, application of CD-GFD serum resulted in the presence of OC from HD PBM after a typical 14 days [56]. In the second study, HD PBM cultured with cytokines and synovial fluid (SF) of RA or pyrophosphate arthropathy (PPA) differentiated into OC after 3 days, while control culture with M-CSF and RANKL, but without SF, generated OC after 5–7 days [68].

The situation concerning rapid one-week differentiation is less clear when it comes to the induced osteoclastogenesis performed on precursors originating directly from patients suffering from RA or AS. Osteoclasts generated from RA PBM under the effect of TNF-α were detectable after 10 days, but not after 7 days [48]. Perpétuo et al. [14,15,28] and Ma et al. [69] have independently observed no OC formation after 7 days of RA or AS PBM induction with M-CSF and RANKL. Others, on the other hand, report successful differentiation of RA PBM [19,20] or AS PBM [18] into OC already after 8 days. This discrepancy may be of a technical nature (Ikić et al. [19] counted OC as cells with 2 or more nuclei, while Ma et al. [69] counted cells with 3 or more nuclei), but perhaps it may also be caused by differences in patient cohorts, as RA patients in the study of Ikić et al. [19] were generally older, had higher disease duration, much higher inflammatory markers (C-reactive protein and erythrocyte sedimentation rate) and higher disease activity score-28 than those in the study from Ma et al. [69]. These comparisons suggest that rapid one-week culture might be a very useful tool for distinction between patients with the same diagnosis but different disease progression.

In RA, PsA and osteoarthritis (OA), results acquired from long culture lasting 3 weeks can differ considerably; though the increased OC differentiation in comparison to control is expected, results seem to be contradictory. Sometimes, patients’ PBM differentiate under the effect of exogenous RANKL and M-CSF into higher OC number than HD PBM after 2 weeks [24,25,70] or 3 weeks of culture [35,36] and other times show no difference after 2 weeks [13,30] or 3 weeks [32]. In AS, however, a remarkable change in dynamics of osteoclastogenesis was noted when increased OC differentiation from AS PBM compared to HD PBM has been observed in one-week long culture [18], no increased OC numbers were found between AS and HD after 2 weeks of culture and subsequently higher differentiation from HD PBM was present after 3 weeks [15]. It needs to be emphasized that increased differentiation from HD PBM in comparison to AS PBM after 3 weeks is not an isolated observation [16].

Although the issue of three different approaches, osteoclastogenesis induced, spontaneous and co-cultures, will be discussed in detail later, it is necessary to state already here that in the case of spontaneous osteoclastogenesis and co-cultures, the length of cell culture probably does not affect the resulting number of OC formed. The results quite consistently show more OC generated spontaneously in disease than in healthy state regardless of culture length (e.g. [30,32,[70], [71], [72]]) and higher differentiation in presence of patient material versus HD in co-culture (e.g. [31,73,74]).

In summary, examples described above show that assessing dynamics of OC differentiation is a useful and important experimental method. This is especially true concerning comparison between arthritic patients and HD or among patients with a specific diagnosis themselves. In the future research, assessment of osteoclastogenesis at more time points could shed light on some important disease-related underlying biological mechanisms, such as different propensity to disease progression and manifestations between patients.

#### 4.3.1 Osteoclast activity at various time points and its concordance with osteoclast numbers

Osteoclastic resorption is a hallmark of function of cultured OC. The activation of fused OC into resorbing cells is a delicate process requiring characteristic polarization of plasma membrane and creating a sealed acidified environment between OC membrane and bone tissue [75]. The triggers of this process are not known at the moment, however, proto-oncogene tyrosine-protein kinase Src and αvβ3 integrin are necessary actors [76,77]. On the cellular level, the fusion process seems to be directly related to the resorptive function in a way that having more nuclei is favorable for OC, because the resorbed surface area rises linearly with the number of nuclei [41,78,79]. The importance of fusion process is further underscored both in pathology and physiology by findings that the expression of gene coding for dendrocyte expressed seven transmembrane protein, which is related to fusion, is intensified by genetic risk variant for Paget's disease of bone [80], and that human aging is accompanied by rise of expression of this gene, together with an increase in serum bone resorption markers [41].

In research of human chronic inflammatory diseases, OC numbers are often consistent with total resorption, which is higher in RA versus HD PBM [25,69], in RA versus OA SFM [81], in PsA versus HD PBM [26,71,82,83] and in CD versus HD PBM [84]. Good concordance has been reported in comparison of erosive versus non-erosive RA, where both differentiation and resorption were higher in erosive RA PBM [69]. Also studies testing the effects of inflammatory molecules and treatments have provided a handful of reports that resorption correlates with differentiation, as expected; evidence exists both under condition when OC formation was stimulated [48,85,86], and when it was suppressed [87,88].

The concordance between the number of 10.13039/100003224OC formed *in vitro* and the rate of resorption was confirmed not only in the induced 10.13039/100003224OC differentiation (as evidenced in examples above), but also in spontaneous osteoclastogenesis and in co-cultures: HD PBM differentiated spontaneously into low 10.13039/100003224OC number and resorbed negligible amount of bone surface compared to PsA PBM [71]; both HD PBM differentiation and resorption decreased in the presence of HD MSC when compared to AS MSC co-culture [66] and further, 10.13039/100003224OC differentiation and resorption were supported in identical manner under the influence of RA T-cells [67]. Therefore, it seems that, in general, the concordance between differentiation and resorption can be expected, regardless of the chosen *in vitro* approach.

Nevertheless, as already implied, there is a considerable amount of research coming to the conclusion that resorption rate does not always correspond to OC numbers. This could be caused by inability of some of the fused OC to turn into resorbing cells. Indeed, it was already described that OC with the ability to resorb can be characterized by high gene expression in their nuclei, while nuclei in non-resorbing OC maintain low activity [78]. Currently, the causes of the altered activity of the nuclei of fused OC are not known, just as it is not known to what extent this mechanism is applied in human pathologies, or possibly what other mechanisms may be involved. Yokota et al. [27] have determined that in RA PBM, TNF-α and IL-6 promote differentiation in a RANKL-independent manner, but the resorptive activity of such OC is low. Interestingly, while OC numbers differentiated under the effect of RANKL correlated negatively with bone mineral density, the TNF-α and IL-6 cytokines induced OC counts positively correlating with modified total Sharp score. This could indicate that – at least in inflammatory arthritis – OC generated by the “classical” pathway via RANKL are involved in bone loss differently than OC generated under the influence of TNF-α and IL-6.

Dickerson et al. [89] have informed that conditioned media from synovial fibroblast cell cultures of RA and 10.13039/501100006728PPA patients support differentiation of HD PBM into more 10.13039/100003224OC than identical media originating from cells of OA patients, but there is no significant difference in the effect on resorption. When comparing differentiation and resorption between HD and OA PBM, only 1.25 times more OC were formed from OA PBM than from HD PBM, whereas resorption was even 4 times higher in OA [36]. In the induced osteoclastogenesis of RA and HD PBM, no difference was found in total OC numbers between RA and HD at any time point of the 1–3 weeks long culture, but resorption was significantly higher after 2 and after 3 weeks in RA [14]. These results are supported by a previously dated finding where RA PBM differentiated into the similar 10.13039/100003224OC numbers as HD PBM, but with a 3 times higher resorption potential [13].

In case of PBM originating from AS patients, these cells have been shown to differentiate after 3 weeks into 50 % lower OC numbers than HD PBM; surprisingly, they were able to resorb the same amount of bone surface [15]. Taking into account presence/absence of ankylosis, osteoclastogenesis from PBM of patients with ankylosis of sacroiliac joint gains more OC than from PBM of subjects without ankylosis, although the resorption is comparable between the both groups [18]. These results may suggest that resorption capacity of differentiated OC changes together with AS progression.

Lastly, assessment of resorption capacity might be very important for the correct choice of treatment, as the drug may have a different effect on differentiation and a different effect on resorption. Resorption can be hit much harder than differentiation – potent inhibition of resorption but limited effect on differentiation was reported for tumor necrosis factor-inducible gene 6 protein in *in vitro* models of arthritic diseases [90]. In addition, when we talk about pathological osteoclastogenesis, treatment can have different outcomes in different diseases: When comparing RA and AS patients, both treated by TNF-α inhibitor infliximab, OC differentiated from PBM of RA patients had lower resorption capacity than those derived from AS subjects [91]. Over the course of appointments during the 6 months of medication, resorption by differentiated PBM remained higher in AS compared to RA, while after 6 months of medication both groups presented with similar inhibition of *in vitro* resorption, suggesting earlier response to medication in RA than AS patients [91].

Gengenbacher et al. [91] and Perpétuo et al. [14,28] have similarly shown that after 6 months of treatment of RA patients, OC in 3-week culture have a reduced resorptive capacity compared to those before treatment. Perpétuo et al. [14,28], however, also assessed resorption after 14 days: Data indicates that it does not get to desired “normal” levels at this point of culture and that 3 weeks are needed to see effects of medication in an *in vitro* resorption model. This suggests that not only the level of resorption, but also dynamics of resorption, which has not been extensively studied so far, could be of a great research interest. All in all, as shown by the examples above, assessment of 10.13039/100003224OC activity by resorption assay is an important aspect of *in vitro* research and should always be considered in order to support/confront data obtained by evaluating the number of differentiated 10.13039/100003224OC.

### 4.4 Other options for identifying osteoclasts and assessing their activity and function

There are several other overlooked aspects of OC biology. The following paragraphs will focus namely on OC apoptosis, number of nuclei per OC (nuclearity) and resorptive activity per osteoclast. Measurements of gene and protein expression will not be discussed further, as the exact molecular basis of osteoclastogenesis is not the aim of this review.

Regarding the propensity to apoptosis, the *in vitro* differentiation resulted into higher OC numbers in RA and OA than in HD, and in addition, patients' OC were shown to be less apoptotic than normal “healthy” OC [35,36]. This combination inevitably increased the predisposition to greater bone resorption. Recent study on RA showed that *in vitro* differentiated OC derived from RA patients with progression into erosive arthritis are more resistant to apoptosis [17]. In contrast to that, OC numbers differentiated in AS were lower and the cells were also less apoptotic than HD OC [16]. One can speculate that impaired apoptosis in AS OC compensates for the reduced production of bone-resorbing cells, and the differences in the level of apoptosis may be related to the unique etiology of AS. Thus, osteoclast apoptosis could help explain specific differences in the progression of rheumatic diseases.

OC nuclearity is usually viewed just as a defining morphological criterion for distinguishing OC and only few perceived its informational potential. Animal studies revealed that nuclei within one OC have identical transcriptional activity [78] and that number of nuclei correlates with overall OC activity [79]. Concerning human diseases, similar OC numbers differentiate from SLE PBM as from HD PBM, and these OC show comparable nuclearity. Nonetheless, medication of SLE patients with mycophenolate mofetil results in decreased number and nuclearity of their *in vitro* generated OC, suggesting that activity is reduced as well [92]. RA PBM were reported to differentiate into more OC than HD PBM; an increase in the number of OC with 10 or more nuclei was mainly responsible for this, while the number of OC with 3–5 nuclei and 6–9 nuclei did not differ significantly between groups [35].

Perpétuo et al. [14] did not observe *in vitro* increased number of OC or number of resorption pits in RA compared to HD cells; they noticed, however, the higher resorption rate per osteoclast (OC nuclei were not analyzed). This indicates that resorbing OC in RA are more active than in controls. A similar effect was also seen in AS OC [15]. Furthermore, *in vitro* generated OC from RA patients on TNF-α inhibitor medication presented with lower resorption per cell than OC from patients before treatment [28]. Comparably, Allard-Chamard et al. [17] found that individual OC differentiated from RA PBM resorbed amount of surface area comparable to those from HD PBM, but were about 2-fold less apoptotic after 3 weeks, suggesting that RA OC would have higher resorptive capacity when assessed later. These studies point to the possibility that changes in numbers of highly active OC (with or without a difference in total number of osteoclasts) could be important in the etiology of inflammatory arthritis.

## 5 Main approaches for studying *in vitro* genesis of osteoclasts

As mentioned earlier in the Introduction and shown in Fig. 2, three approaches can be employed when studying the role of OC differentiation under *in vitro* conditions. Their major characteristics are summarized in Fig. 2. Here we will focus on the use of individual approaches for the *in vitro* study of bone resorption in rheumatic autoimmune diseases in the context of their specificities.

### 5.1 Induced versus spontaneous osteoclastogenesis

Recently, the question of whether to artificially stimulate OC precursors with exogenous RANKL and M-CSF has come to the fore. In the review of Salamanna et al. [93], it is shown that in many diseases including rheumatic diseases and bone-metastasizing cancer, the mononuclear cells of patients differentiate spontaneously into OC. It seems that it is precisely because of this special ability that their cells can often be distinguished from healthy donor cells, from cells obtained from patients with other diseases or even at a different stage of the disease. To give an example, mononuclear cells from patients with PsA have been shown to differentiate spontaneously into higher OC numbers than those from psoriasis vulgaris patients and/or HD cells [30,32,70,71]. The same seems to take place concerning RA monocytes versus HD monocytes [72,86].

Interestingly, when induced osteoclastogenesis is applied, some studies reveal comparable number of differentiated OC between PsA and HD [20,30,32], RA and HD [17,94], or even between patients with different diagnoses, e.g. RA and OA [35,81] or RA and PsA [20]. As already implied, in some cases, the over-stimulation with artificial cytokines may hide the biological processes that might be taking place *in vivo* and that would be otherwise revealed by spontaneous osteoclastogenesis. Such assumption can be further supported by the fact that induced *in vitro* osteoclastogenesis with “sub-optimal” RANKL doses has been used to uncover some differences in osteoclastogenic potential between patients and HD [13,56]. This means that spontaneous osteoclastogenesis can be more useful in situations where we want to assess the biological processes as closely to the *in vivo* system as possible. It may be particularly helpful in evaluation of natural osteoclastogenic capacity of precursors derived from certain individuals with a specific diagnosis, therefore better for distinguishing patients with various forms of illness or patients from HD subjects, for correlations between biological and disease markers and construction of predictive and screening models.

Induced osteoclastogenesis, on the other hand, may represent the only approach with a fully controlled and reproducible environment, and as such it is very useful in *in vitro* testing of the effect of specific molecules and agents on OC formation and function. It can also make it easier to compare the outcomes of different studies. For example, Ikić et al. [19] found that in the presence of RANKL and M-CSF, more than 2-fold higher number of osteoclasts differentiated from PBM of RA patients compared to PsA patients. In comparison, research from Marton et al. [20] revealed comparable OC differentiation between RA and PsA with a slight trend towards higher differentiation in PsA. Both teams used equivalent methods and had similar number of participants, however, in Ikić et al. [19], RA and PsA patients had similar duration of disease exceeding ten years and only 50% of RA patients were on disease-modifying anti-rheumatic drugs (DMARD) treatment, while in Marton et al. [20], PsA patients had duration only over 5 years, and 95% of RA patients were on DMARD treatment. Thus, the discrepant findings could possibly be attributed to treatment or disease duration, but this interpretation should be taken with caution.

Perhaps even more intriguing is the induction of osteoclastogenesis from the precursor cells of patients suffering from AS, disease characterized by the simultaneous occurrence of both processes, pathological bone neo-formation and resorption. When serum acquired from patients in early stages of AS is used in culture for HD PBM differentiation, it possibly exerts its pro-osteoclastogenic effect as it stimulates OC differentiation significantly more than healthy serum [95]. In contrast to that, when PBM from AS patients in later stages are differentiated, they have been repeatedly shown to produce less OC than HD PBM [15,16]. This could possibly be interpreted so that AS PBM have limited responsiveness to osteoclastogenic stimuli even when they come from the internal environment that is overall stimulating towards differentiation into OC. It would be interesting to find out what role the RANKL-RANK axis plays in AS, and responsiveness to various RANKL concentrations might help to clarify that. This example shows that induced osteoclastogenesis may be also very useful when assessing one of the aspects of pathological osteoclastogenesis – an altered reaction of OC precursors to stimuli.

### 5.2 Cell co-culture

Osteoclast precursors come physiologically into contact with a variety of cells, including lymphocytes circulating in blood, fibroblasts/FLS or mast cells present at sites of inflammation, or mesenchymal stem cells/bone marrow stromal cells (BMSC). All of these have been confirmed to play a role in pathological osteoclastogenesis, at least when it comes to the *in vitro* model.

Regarding lymphocytes, RA memory CD19^+^ and regulatory CD19^+^CD24^hi^CD27^+^ B-cells support 10.13039/100003224OC differentiation more than the same cells derived from HD or OA individuals [33,96]. CD3^+^ T-cells of RA patients greatly stimulate OC generation in co-culture with BMM or PBM [67,73]. In contrast, co-culture of HD CD3^+^ T-cells and PBM leads to formation of only few OC [73]. Similarly, OC differentiation is ten times higher in the presence of CD3^+^ T-cells derived from CRD patients than those from HD [97]. In one case, even NK cells were shown to support 10.13039/100003224OC formation [98].

Fibroblasts coming from RA patients are able to support 10.13039/100003224OC generation when pre-stimulated *in vitro* with factors such as TNF-α and IL-1β [99], Th17 cytokines [59,100,101] or macrophage inhibitory factor [102]. In case of toll-like receptor ligands, the effects can be both stimulatory [31,103] and inhibitory [104]. FLS share common characteristics with fibroblasts and RA FLS have been shown to support 10.13039/100003224OC generation when pre-stimulated with TNF-α and IL-17 [[105], [106], [107]], vascular endothelial growth factor [108] or CCAAT/enhancer-binding protein β [62]. Lastly, mast cells are also able to stimulate OC differentiation in RA [60].

The MSC/BMSC have a dual role in physiological osteoclastogenesis, which resides in the production of pro-osteoclastogenic signals, but also anti-osteoclastogenic cytokine osteoprotegerin [109]. MSC have been used in co-cultures with OC precursors to study diseases with defects in new bone formation. AS MSC exert a strong inhibitory effect on differentiation when cultured together with HD PBM in the presence of RANKL and M-CSF [66].

It is clear that factors produced by mentioned cells are one of the culprits of their regulatory effect in OC differentiation. When conditioned media from PsA PBM, RA FLS and PsA skin-derived keratinocytes and T-cells are used, they support 10.13039/100003224OC differentiation from healthy PBM [26,61,71,74]. Similarly, conditioned media from RA synovial fibroblasts or peripheral blood mononuclear cells also lead to osteoclast differentiation from healthy PBM [110,111]. Nonetheless, the importance of direct cell-cell contact may not be overlooked, as for example in case of mast cells in RA, direct contact is necessary for their stimulatory action [60]. In summary, cell or supernatant co-culture experiments are very useful for elucidating the roles of specific cells and molecules in pathologies, as they can reveal cell types whose inhibition *in vivo* may benefit a patient with the disease.

## 6 Conclusion and future directions

As *in vitro* osteoclastogenic research grew over time, there are now several methods and three main approaches by which *in vitro* osteoclastogenesis can be assessed. This broad range of methods and approaches available for *in vitro* osteoclastogenesis should not be perceived as mere technicalities or burdens, but rather as opportunities for studying unresolved aspects of (not only) autoimmune diseases. In this review, we have shown how the choice and use of individual methods and approaches can help explain discrepancies between the outcomes of studies focusing on the same issue, but more importantly, may also contribute to the elucidation of the underlying biological mechanisms of the pathology under study. Main limitation of this review is that it was not possible to conduct meta-analysis due to heterogeneity of research objectives and methods of the included studies. Therefore, only qualitative synthesis using a descriptive approach was achievable. Still, to the best of authors' knowledge, this is the most extensive investigation on the topic to date.

The most important deductions that can be drawn from the provided information synthesis about use of different methods and approaches in the *in vitro* osteoclastogenic research of autoimmune diseases are:

● OC precursors originating from PBM and SFM may pose different risks and/or treatment opportunities with respect to particular pathology (as shown by their different involvement in RA and PsA, and different reactions to inhibitors).
● Osteoclastogenesis in specific cell subtypes may be linked to a particular form of disease, disease progression and/or response to treatment (as confirmed for example on immature dendritic cells and myeloid-derived suppressor cells in RA).
● Faster dynamics of *in vitro* OC generation seems to be associated with higher disease activity and burden (as revealed e.g. by the effect of sera obtained from active CD patients compared to sera from GFD-treated CD subjects, or by higher osteoclastogenic potential of OC precursors from highly active RA).
● Seemingly, even when the number of OC generated *in vitro* is comparable between precursors of diseased and healthy origin, the general recognizable feature of OC differentiated from patient precursors is their higher activity (when assessed by resorption per osteoclast, nuclearity, or lower propensity to apoptosis). Next to that, higher OC activity may be also linked to higher disease activity and burden.
● Spontaneous osteoclastogenesis may better reflect *in vivo* conditions than induced osteoclastogenesis; moreover, it is an important tool for distinguishing the diseased (patient) versus healthy origin of the donor cell/serum.
● Induced osteoclastogenesis is useful in cases where a controlled and reproducible environment is desirable (such as testing the effect of molecules or agents under study). However, induced osteoclastogenesis performed in presence of only low RANKL concentrations may fulfill the same role without the risk of hiding biological effects of tested factor (shown e.g. in different connection of RANKL-dependent and TNFα and IL-dependent osteoclastogenesis to clinical factors).
● It is also important to recognize that induced osteoclastogenesis, though the most frequently applied approach, often in its basic form (e.g. without any methodological modifications such as the use of various cytokine concentrations, assessment of osteoclastic differentiation dynamics, nuclearity of differentiated osteoclasts, resorptive activity per cell), is not always able to provide relevant answers concerning bone metabolism dysbalance and etiopathogenesis of autoimmune diseases.
● Cell co-culture is a great tool for identifying cell types involved in pathological osteoclastogenesis; it also indicates which cell type inhibition might be useful in a particular disease or form of disease.

We also suggest the following directions for the use of *in vitro* osteoclastogenesis, which in our opinion could help in future research of autoimmune diseases:

● Finding correlations between clinical parameters and *in vitro* osteoclastogenesis is a critical step for its possible use in clinical research. Therefore, a wider use of the approach of spontaneous osteoclastogenesis together with the assessment of the OC formation dynamics and OC activity is needed in the future research. Larger cohorts of patients should also be collected.
● Characteristics of patient *in vitro* osteoclastogenesis seem to change after therapy (e.g. immune therapy in RA or medical nutrition therapy (GFD in CD)). More knowledge is needed about particular changes after both, successful therapy and therapy with lower effect or effect different than desired, as this could explain the latter and therefore lead to better options in precision medicine and treatment in the future.
● There seem to exist some similarities between AS and other rheumatic diseases (higher OC activity *in vitro*, pro-osteoclastogenic internal environment) but also some distinctions (lower response of precursors to osteoclastogenic stimuli, different dynamics of *in vitro* osteoclastogenesis). Hence, more focus should be placed on the study of *in vitro* osteoclastogenesis in AS, as it might help to explain not only specific etiopathology of AS, but also the course of inflammatory osteoclastogenesis in general.
● Compared to rheumatic diseases, *in vitro* osteoclastogenesis has seen much less use in enteropathies. In CD, the risk for osteopenia/osteoporosis development is increased despite the dietary intervention [112]. Next research should include lessons about the dietary and anti-inflammatory pharmacological intervention on OC development/osteoclastogenesis in enteropathic diseases.
● Lastly, no comprehensive review regarding osteoclastogenesis research using *in vivo* mouse (osteoclastogenic) models, simultaneously investigated *in vitro*, is currently available. Such review would be a valuable follow-up to this paper and could greatly benefit future transition of osteoclastogenesis disease models into clinical practice.

It is becoming clear that research of the osteoclastogenesis process could have even greater potential than previously thought. Its use in elucidating underlying causes of bone pathologies is slowly shifting towards future use in precision medicine (i.e. determining groups of patients at highest risk, finding best possible treatment), screening among patients or even among the general aging population. Therefore, good understanding of possibilities and limitations of various research methods and approaches is now more important than ever. Hopefully, this understanding will help facilitate and accelerate translation of basic research results into clinical practice, which could immensely help with ever increasing health concerns that autoimmune diseases and bone health in general undeniably represent.

## Funding

This work has been supported by the 10.13039/100007397Charles University Research Centre program No. 204069 and by the 10.13039/100009647Ministry of Health, Czech Republic under Project for the conceptual development of research organization 00023728.

## Data availability statement

No data was used for the research described in the article.

## CRediT authorship contribution statement

**Patrik Skubica:** Writing – review & editing, Writing – original draft, Investigation, Formal analysis, Conceptualization. **Marketa Husakova:** Writing – original draft, Writing – review & editing. **Pavlina Dankova:** Writing – review & editing, Writing – original draft, Supervision, Conceptualization.

## Declaration of competing interest

The authors declare that they have no known competing financial interests or personal relationships that could have appeared to influence the work reported in this paper.
